# The Combination of Stereotactic Body Radiation Therapy and Immunotherapy in Primary Liver Tumors

**DOI:** 10.1155/2019/4304817

**Published:** 2019-04-28

**Authors:** Malek Kreidieh, Youssef H. Zeidan, Ali Shamseddine

**Affiliations:** ^1^Division of Hematology and Oncology, Department of Internal Medicine, American University of Beirut, Beirut, Lebanon; ^2^Department of Radiation Oncology, American University of Beirut Medical Center, Beirut, Lebanon

## Abstract

Treatment recommendations for primary liver malignancies, including hepatocellular carcinoma (HCC) and cholangiocarcinoma (CCA), are complex and require a multidisciplinary approach. Despite surgical options that are potentially curative, options for nonsurgical candidates include systemic therapy, radiotherapy (RT), transarterial chemoembolization (TACE), and radiofrequency ablation (RFA). Stereotactic Body Radiation Therapy (SBRT) is now in routine use for the treatment of lung cancer, and there is growing evidence supporting its use in liver tumors. SBRT has the advantage of delivering ablative radiation doses in a limited number of fractions while minimizing the risk of radiation-induced liver disease (RILD) through highly conformal treatment plans. It should be considered in a multidisciplinary setting for the management of patients with unresectable, locally advanced primary liver malignancies and limited treatment options. Recently, the combination of immunotherapy with SBRT has been proposed to improve antitumor effects through engaging the immune system. This review aims at shedding light on the novel concept of the combination strategy of immune-radiotherapy in liver tumors by exploring the evidence surrounding the use of SBRT and immunotherapy for the treatment of HCC and CCA.

## 1. Introduction

### 1.1. Primary Liver Tumors

Primary liver cancer is the seventh most common cancer world-wide, with around 841,080 newly diagnosed cases in 2018 [1]. It is the third leading cause of cancer deaths in the world, with an estimated 781,631 liver cancer deaths occurring in 2018 [1]. It is also the fifth largest contributor to cancer mortality in the United States [2]. Although patients diagnosed at early stages have a relatively good prognosis, the majority of patients are diagnosed at later stages. The 5-year survival rate for all Surveillance, Epidemiology, and End Results (SEER) stages combined is 18%, and it drops to 2% in patients presenting initially with late stage disease [2, 3]. The two most common subtypes of primary liver tumors are HCCs that arise from hepatocytes and intrahepatic cholangiocarcinoma (IHCs) that arise from epithelial cells of the intrahepatic bile ducts [4].

### 1.2. Hepatocellular Carcinoma: Epidemiology and Prognosis

HCC accounts for 75 to 85% of primary liver cancers world-wide [1]. Its prevalence is highest in Eastern and Southern Asia and among males [5]. Recently, although the incidence has been declining in high-risk regions, the incidence in lower-risk areas including India, Europe, and North America is on the rise as rates of hepatitis C, obesity, and diabetes continue to increase. For instance, it has doubled from 2.6 to 5.2 per 100,000 populations over the period between 1990 and 2014 [6, 7].

HCC is the second most frequent cause of cancer death in men and the sixth leading cause of cancer death in women [1, 8]. Although surgical resection, liver transplantation, and ablation offer a potential for cure, only 20% of patients with HCC are suitable for primary surgical management at the time of diagnosis [9, 10]. The remaining 80% are diagnosed at advanced stages when curative treatments become nonfeasible [11, 12]. In fact, most patients with HCC often present with locally advanced, unresectable disease, when the tumor has already extended or invaded major vasculature. The absence of effective therapies in such cases contributes to the poor prognosis of HCC, with a 5-year survival rate and a median overall survival (OS) that are less than 5% and 1 year, respectively [13–15]. Patients with advanced HCC are therefore offered nonsurgical approaches such as chemotherapy, targeted therapy, immunotherapy, TACE, RT, or percutaneous ethanol injection (PEI) [16–19]. Not only does the dismal prognosis of HCC patients stem from the advanced stage at presentation, but also it arises from high recurrence rates. In fact, nearly 80% of tumors recur 5 years following hepatic surgery [20].

### 1.3. Intrahepatic Cholangiocarcinoma: Epidemiology and Prognosis

The pathogenesis of IHC seems to be related to chronic inflammation and the resulting oxidative stress created in bile ducts [21]. IHC constitutes around 3% of gastrointestinal cancers [22]. It is the second most common primary hepatic malignancy in the United States following HCC, with around 5000 newly diagnosed cases per year [1]. The relative incidence was higher in men than in women over the period from 2008 to 2012 [22]. Several epidemiological studies show that while the incidence of extrahepatic cholangiocarcinoma (EHC) has decreased or stabilized, that of IHC continues to increase and has doubled among Asians as compared to African-Americans and Caucasians [22, 23].

The 5-year survival in IHC patients is less than 10%. The dismal prognosis is due to advanced stages at time of diagnosis, limited treatment options, and very high rates of recurrence and metastases [24]. Surgical resection remains the only potentially curative treatment option and is rarely feasible except in early stages of IHC [25]. Unfortunately, however, less than 20% of patients with IHC are candidates for surgical resection at the time of diagnosis. The remaining 70% have unresectable or advanced diseases requiring systemic therapies such as chemotherapy [26–28]. Such nonoperative therapies have significant limitations and the median survival for patients with inoperable disease remains poor (7 to 12 months). Even among patients who are surgical candidates, recurrence rates are as high as 52%, and 5-year postresection survival rates range from 8% to 44% [23, 27–31]. Whether primary or recurrent, most patients survive about 6 months in the absence of any treatment [23, 27, 29, 30, 32].

## 2. Management: Evolving Paradigms in Immunotherapy and Radiotherapy

Treatment recommendations for primary liver tumors are complex and require a multidisciplinary approach. Despite surgical options that are potentially curative, options for nonsurgical candidates include systemic therapy (immunotherapy, tyrosine kinase inhibitors [TKIs], and chemotherapy), external-beam radiation therapy (EBRT), TACE, and percutaneous tumor ablation ( [RFA], microwave ablation [MWA], PEI, and cryotherapy). Here we focus on evolving RT and immunotherapy approaches.

## 3. Radiotherapy

Historically, EBRT has not played a substantial role in the treatment of liver malignancies secondary to the limited tolerance of the whole liver to radiation. Over the past decade, the approach to liver cancer patients has been affected by a paradigm shift that has revolutionized RT [37–39]. Consequently, RT has become the preferred treatment option for inoperable patients with tumors situated near the main portal vein, inferior vena cava, or the hilum of the liver [40]. Such tumors can cause liver failure related to vascular or biliary compromise, and surgical resection is not an ideal alternative given the location.

### 3.1. Radiotherapy and the Immune Response

It is well established that RT has direct cytotoxic effects on cancer cells and can generate a robust antitumor immune response through effects on both the tumor and its microenvironment. This occurs via a variety of mechanisms including enhanced tumor antigen presentation and upregulated major histocompatibility complex (MHC) class I expression [41]. The high doses of radiation used in SBRT increase tumor-cell lysis at the level of the localized treatment site and release tumor-associated antigens (TAA) in the process. The released TAAs are taken up by professional antigen presenting cells (APC), including dendritic cells (DC) and macrophages [42, 43]. Proinflammatory cytokines can then activate the APCs that migrate in turn to tumor-draining lymph nodes. Here, CD8+ cytotoxic T-cells are activated to provide antitumor immunity [44]. In addition to enabling the mobilization of T-cells against cancerous cells with the help of released TAAs, radiation results in the translocation of calreticulin (CRT) to the tumor-cell surface [45, 46]. This serves as a signal to activate macrophages and DCs which internalize CRT-expressing tumor cells.

Emerging clinical data suggest that RT may have systemic effects that go far beyond the locally irradiated target [47, 48]. Such abscopal effects refer to the ability of radiation delivered to a local site to minimize or eradicate metastases at distant sites, outside of the treatment field [49]. This nonspecific eradication of distant tumors and metastases can be accounted for by the systemic increase in the levels of proinflammatory cytokines and chemokines released from both the immune cells and tumor tissues, following exposure to radiation [43].

### 3.2. Stereotactic Body Radiation Therapy

SBRT is a highly specialized form of EBRT that delivers high doses of precisely targeted radiation in a few fractions to a tumor and minimizes radiation dose to adjacent normal tissue structures [50, 51]. It maximizes the cell-killing effect on the target, while at the same time minimizing injury in adjacent normal tissues. This hypofractionated image-guided RT is typically utilized for small tumors that require precise targeting. It is made possible by major improvements in patient immobilization, positioning accuracy, organ motion assessment, and radiation planning techniques [52].

SBRT is now in routine use for the treatment of lung cancer, and there is growing evidence supporting its use in primary liver tumors. Data reveal 1-year local control (LC) rates exceeding 90% following the use of SBRT in HCC [53] and hepatic metastases [54]. This has rendered SBRT the focus of many studies that assess its safety and efficacy in primary hepatic tumors [55]. The use of high-dose ablative radiation is currently under evaluation in HCC in a phase III trial (NCT01730937) by the Radiation Therapy Oncology Group.

### 3.3. Stereotactic Body Radiation Therapy versus Percutaneous Tumor Ablation

Percutaneous tumor ablation, typically by RFA or MWA, is usually performed in patients with early stage, unresectable HCC. A retrospective study from the University of Michigan highlighted the relative efficacy of using SBRT as compared to percutaneous tumor ablation in patients with unresectable HCC [56]. Freedom from local progression (FFLP) rates at 1 and 2 years was lower with RFA than with SBRT for all tumors, whether less than or more than 2cm in size. The rates of grade 3 or higher acute toxicity were higher with RFA (11%) than with SBRT (5%). An ongoing trial at the Durham VA Medical Center is currently comparing the use of SBRT and MWA in surgical candidates who decline surgery or nonoperative, early stage HCC cases [NCT03402607].

### 3.4. Stereotactic Body Radiation Therapy versus Transarterial Chemoembolization

A large single-institution comparison of TACE and SBRT outcomes was performed on 209 patients with small HCC tumors (2.3-2.9cm) [57]. 84 patients with 1 to 2 tumors underwent TACE to 114 tumors, and 125 patients with 1 to 2 tumors underwent SBRT to 173 tumors during the period from 2006 to 2014. While no OS differences were noted, 2-year LC rates were higher with SBRT than with TACE (91% versus 23%). In addition, grade 3+ toxicity rates were higher in the TACE arm than in the SBRT arm (13% versus 8%). This suggests that SBRT can be used as a safe alternative to TACE.

Whereas TACE remains the most common locoregional treatment to serve as a bridging modality in HCC patients undergoing liver transplantation, the best one remains unclear. In a randomized phase II trial, 29 HCC patients with Child-Pugh Class (CTP) A/B liver cirrhosis who were planned for liver transplantation were randomized to either SBRT or TACE from 2014 to 2016 [58]. 12 patients received SBRT for a median total dose of 45Gy delivered over 5 fractions, and 15 patients received 2 TACE treatments. SBRT was shown to reduce hospitalizations and to be equally effective in TACE as a bridge to liver transplantation. The ongoing TRENDY trial [NCT02470533] is based on the hypothesis that the time to progression is more favorable after SBRT than after TACE in HCC patients ineligible for surgery or RFA. Results are expected to be available in April 2020. SBRT, therefore, represents a noninvasive, potentially curative modality that can be utilized in the definitive treatment of patients with HCC and as a bridge for patients potentially eligible for transplantation. While the 2016 NCCN guidelines listed EBRT as a viable option for the treatment of advanced HCC, current 2018 NCCN guidelines implement the role of SBRT in both resectable and nonresectable cases planned for transplantation [59].

In order to evaluate the combination of SBRT and TACE, a retrospective study of patients with HCC that are larger than 3cm in size was conducted [60]. Patients treated with SBRT following TACE experienced a median survival that is 13 months longer than that of patients treated with TACE alone. They tolerated SBRT well with no instances of significant morbidity being noted. The favorable survival outcomes resulting from the combination therapy support the notion that the strengths and weaknesses of SBRT and TACE are complementary. The improved survival mainly stems from improved LC rates and local recurrence rates in the combination group. Many ongoing studies are currently evaluating the combination of TACE with SBRT [NCT01918683, NCT02507765, NCT02513199, and NCT02794337] and comparing SBRT to TACE [NCT02182687 and NCT02470533].

### 3.5. Studies Evaluating the Use of Stereotactic Body Radiation Therapy for Hepatocellular Carcinoma and Intrahepatic Cholangiocarcinoma

Despite its relatively recent adoption into clinical practice, SBRT use in liver tumors was first described in the early 1990s in a pilot study by Blomgren et al. [61]. Nine patients with HCC and one patient with IHC were treated with one to three fractions of 5 to 15Gy. Objective response (OR) rates were 70%, with around 50% of tumors having been shrunken or disappeared at time of evaluation and around 80% of tumors having not progressed on follow-up after 1.5 to 38 months. A number of retrospective studies and large single-institution phase I and II prospective trials evaluating the use of SBRT in the treatment of primary liver tumors followed.

One retrospective study by Sanuki et al. included 185 liver tumors ranging in diameter from 0.8 to 5cm and used SBRT doses ranging from 30 to 40Gy in 5 fractions [62]. 3-year LC and OS rates were 91% and 70%, respectively.

A phase I trial included 41 patients (31 HCC and 10 IHC) with unresectable tumors that have a median liver tumor size of 173mL [11]. After completing an SBRT treatment with a median dose of 36Gy in 6 fractions, the median survival for all patients was 13.4 months. 1-year survival and 1-year infield LC rates were 51% and 65%, respectively. No RILD or treatment-related grade 4 or 5 toxicities were noted within the first 3 months after treatment. The promising results from this phase I trial laid the foundation for further phase II and III studies of six-fraction SBRT in this setting.

A phase II trial from Princess Margaret Cancer Centre included patients with CTP Class A disease and used SBRT doses ranging from 24 to 54Gy in six fractions [53]. Results revealed a median OS of 17 months and a one-year LC rate of 87%. Grade 3 or higher toxicities were noted in 36% of patients and primarily consisted of asymptomatic lab abnormalities.

At the University of Michigan, an individualized dose allocation strategy using hyperfractionation was developed for liver cancer treatment and was evaluated by a phase II study. It included 46 IHC patients who were treated with conformal hyperfractionated RT with concurrent hepatic arterial fluorodeoxyuridine. Compared with historical controls, patients had significantly improved OS with a median survival of 13.3 months [63].

Therefore, based on initial promising data, SBRT seems to be a safe, effective, and noninvasive treatment option for carefully selected patients with unresectable tumors that are not amenable to other treatments.

### 3.6. Combining Stereotactic Body Radiation Therapy with Immunotherapy

**Figure 1:**
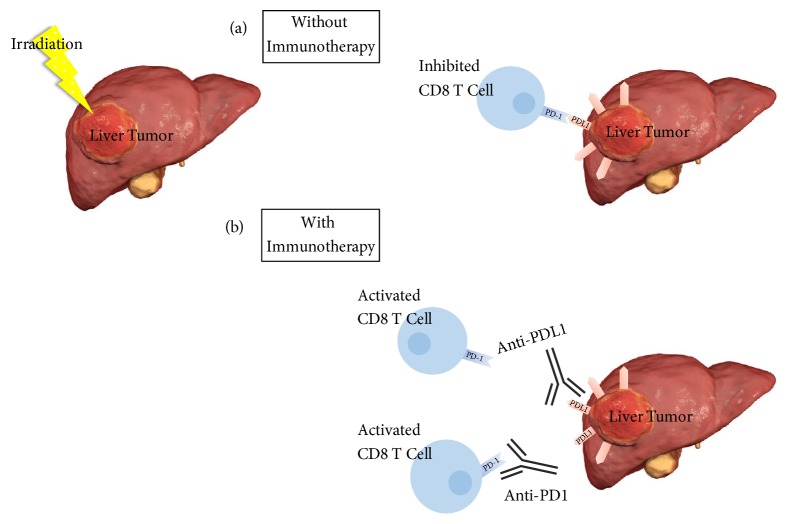
Irradiation of liver tumors with and without immunotherapy. (a) Liver tumors usually acquire radio-resistance through programmed-death ligand-1 (PD-L1) upregulation after radiation since the PD-L1/ programmed-death-1 (PD-1) axis induces CD8 T-cell exhaustion and results in tumor escape from the host immune response. (b) In order to overcome limitations of PDL1 expression upregulation and to reduce the rate of tumor recurrence in primary liver tumors, an immune-based treatment approach targeting PDL1 or PD-1 might be of help in harnessing an immune response to effectively kill liver tumor cells and reduce the rate of tumor recurrence.

Although there is evidence that RT alone provides the necessary signals for the cross-priming of cytotoxic T lymphocytes against tumor antigens, the adjuvant effect of RT appears to be relatively weak, and abscopal responses to RT alone are extremely rare. Also, despite the antitumor effect induced by RT, tumors often develop resistance via immune-escape mechanisms that promote recurrence [64, 65]. In HCCs, for example, frequent resistance to RT is acquired, often resulting in recurrence [66]. This radio-resistance usually occurs through programmed-death ligand-1 (PD-L1) upregulation after radiation [67, 68]. The PD-L1/ programmed-death-1 (PD-1) axis then induces T-cell exhaustion and results in tumor escape from the host immune response, as illustrated in Figure 1.

In order to overcome limitations created by the PD-L1/PD-1 interaction and to reduce the rate of tumor recurrence in primary liver tumors, novel therapies are required. Recent discoveries in tumor immunology, paralleled by technological advances in RT, have provided a promising role for combining SBRT with immunotherapy to augment and sustain the proimmunogenic antitumor effects seen with SBRT alone.

## 4. Immunotherapy in Liver Tumors

Since immune checkpoint inhibitors (ICIs) were first reported in 2010 and 2012, they have translated into a significant OS advantage in comparison to established therapies in metastatic melanomas and nonsmall-cell lung cancer (NSCLC) [69]. Their role is currently being evaluated in some gastrointestinal cancers. Given the background of chronic inflammation in the pathogenesis of many primary liver tumors, the use of immune-based treatment approaches might have a role in releasing the brakes created by the tumor on the immune system [70, 71]. Also, in order to overcome limitations of PD-L1 expression upregulation, an immune-based treatment approach targeting PD-L1 might be of help in harnessing an immune response to effectively kill liver tumor cells and reduce the rate of tumor recurrence. The two most actively studied inhibitory immune checkpoint receptors are cytotoxic T-lymphocyte associated antigen-4 (CTLA-4) and PD-1.

*(1) Anticytotoxic T-Lymphocyte Associated Antigen-4 Agents.* CTLA-4 receptor is exclusively expressed on regulatory T-cells, naïve T-cells, and activated T-cells, and it acts as a regulator of immune cells [72–74]. Through its binding to CD80 and CD86 located on APCs, it promotes immunosuppressive effects of regulatory T-cells [75, 76]. It is currently the second most common checkpoint receptor to be targeted.

The anti-CTLA-4 antibody, Tremelimumab, was evaluated in a phase I trial that included 21 patients with inoperable HCC tumors [77]. 17.6% and 76.4% of patients had partial response (PR) and stable disease (SD), respectively, and 45% of patients experienced SD for more than 6 months. Although the use of Tremelimumab was well tolerated, it has not been approved yet by the FDA. The results of an ongoing phase I clinical trial combining Tremelimumab with RFA or TACE are still pending.

*(2) Antiprogrammed-Death Ligand-1 and Antiprogrammed-Death -1 Agents. *PD-1 receptor belongs to the CD28 superfamily and is expressed on regulatory T-cells, B-cells, and myeloid derived suppressor cells (MDSCs) [78]. It transmits coinhibitory signals and limits tumor-infiltrating lymphocytes (TILs) and T-cell proliferation in peripheral tissues. This results in effective immune resistance in the tumor microenvironment [79, 80]. Most data suggest that TILs are established prognostic markers in melanomas and breast cancers, and a recent study has shown that this applies to liver tumors as well [81, 82]. For instance, the presence of CD8+CD45RO+ TILs in surgical specimens obtained from EHC patients has been associated with prolonged OS [83].

PD-L1 is undetectable in most normal tissues and is inducible by inflammatory cytokines, especially types I and II interferon (IFN) [70]. It is frequently expressed on the surface of tumor cells. One study included tumor tissue samples from 37 patients with CCA and analyzed them immunohistochemically for markers including PD-L1 [84]. Almost 94% of samples were positive for PD-L1. Another study that included EHC tumors showed that 12% of tissue samples and 30% of tumor-associated macrophages were positive for PD-L1 [85]. This increased expression of PD-L1 in CCA cells was shown to be associated with poor prognosis [86]. In another study done on HCC patients by Shi et al., increased PD-1 expression in circulating and tumor-infiltrating CD8+ T-cells was also associated with poor disease progression [87].

Many phase I/II trials have shown promising outcomes with the use of the humanized anti-PD-1 antibody, Nivolumab, in patients with advanced melanoma, lung cancer, and renal cell carcinoma (RCC) [88]. This was followed by several trials evaluating its efficacy in HCC patients. Interim analysis results of a phase I/II trial (CA209-040) were presented at the 2015 ASCO meeting in Chicago, and they were promising considering the poor characteristics of recruited HCC cases [89]. For instance, the use of Nivolumab in a range from 0.1 to 3 ml/kg was associated with a 62% OS rate, a 19% response rate, a 5% complete response (CR) rate, and a 67% disease control rate at 12 months. Also, despite having terminated Nivolumab therapy several months after the attainment of CR, the two patients who attained CR within 3 months maintained this response for longer than 18 months. Such durable responses were observed at all dose levels of Nivolumab in all cohorts.

Results also showed that all participants, whether infected by HBV or HCV or not, encountered tumor size reduction [89]. One patient continued to have reduction in tumor size from around 10 to 2cm over a period of 48 weeks, and another patient had a marked drop in alpha-fetoprotein level from 21,000 to 283ng/mL. Many cases had a large number of multiple HCCs disappear after 6 weeks of therapy.

The report also indicated that Nivolumab monotherapy had a favorable safety profile in HCC similar to that seen in other types of cancer [89]. In fact, a dose-escalation study revealed that Nivolumab can safely be administered up to a dose of 3 ml/kg in HCV- or HBV-infected individuals and a dose of 10 ml/kg in the uninfected group. The only CTCAE grade IV adverse event noted was an elevation in lipase levels. Grade III increases in aspartate amino transferase and alanine amino transferase levels were seen in 11% and 9% of patients, respectively.

Many ongoing trials are assessing the efficacy of combining Nivolumab with gemcitabine/cisplatin on one hand, or Ipilimumab on the other, as a first-line therapy in advanced, unresectable CCA [90]. A single institution study is evaluating the efficacy of combining Nivolumab (n=4) or Pembrolizumab (n=10) with the multikinase inhibitor, Lenvatinib, as a second-line therapy in advanced biliary tract cancer cases who have failed prior anticancer therapy. Results from the interim analysis of the latter study were promising and showed a median PFS of 5 months and an OR rate of 21.4%. Also, 21% (n=3) of patients had PR and 79% (n=11) had SD [91].

A phase II trial evaluated whether testing for mismatch-repair (MMR) deficiencies in treatment-refractory cases of IHC might be of help in identifying those who might benefit from PD-1 pathway blockade [92]. Out of the 4 patients with MMR-deficient, metastatic, and treatment-refractory IHC who were treated with Pembrolizumab, one (25%) had CR and 3 (75%) had SD. In the interim analysis of the phase II KEYNOTE-028 trial (NCT02054806) that evaluated the role of Pembrolizumab in advanced CCA, almost 34% (n=8) of patients with positive PD-L1 expression had PR or SD [93]. It remains unclear, however, whether mutational tumor burden or PD-L1 expression is a better predictive biomarker in CCA.

Ongoing trials will provide us with more details about the role of targeted immunotherapy in primary liver tumors. Antibodies targeting PD-1, PD-L1, or CTLA-4 are expected to be approved and implemented in the setting of HCC and IHC in the very near future.

## 5. Rationale for the Combination of Immune Checkpoint Inhibitors and Radiotherapy

Immunotherapy has emerged as an attractive therapy for refractory cancers. Although results obtained in melanoma and NSCLC patients were beyond what is achievable with conventional therapies, it is expected that such responses will only occur in a subset of HCC or IHC patients who have a high tumor mutational burden [94]. In order to complement the therapeutic effect of immunotherapy in primary liver tumors, there is a need for a combination strategy. Emerging data demonstrate that one strategy to bolster the systemic antitumor immunity in response to immunotherapy is to combine it with RT [95]. Similarly, an abundance of studies suggest that the immunomodulatory effects of RT can be leveraged when combined with immune-based approaches [96].

RT results in the immunogenic death of tumor cells. It primes tumor-specific T-cells and induces the production of IFN-beta by T-cells, thereby enhancing MHC class 1 expression on parental and resistant cancer cells [97]. This restores the responsiveness of resistant tumors to anti-PD-1 therapy. The addition of anti-PD-1 antibodies to RT is therefore expected to promote the proimmunogenic tumor microenvironment.

### 5.1. Synergistic Effects of the Combination Therapy in Preclinical Studies

Many preclinical studies demonstrate a synergistic effect when RT and CTLA-4 or PDL-1 inhibitors are combined [67]. In a study by Yoshimoto et al., mice exposed to the combination of RT and anti-CTLA-4 antibody had improved antitumor immunity and prolonged tumor growth delay (from 13.1 to 19.5 days) when compared to those exposed to RT alone [98]. Vanpouille-Box et al. assessed the use of radiation-induced vaccination in mouse tumor models and observed improved survival following treatment with PD-1 and TGF*β* blockade but not with TGF*β* blockade alone [99]. Exposure of mice with intracranial gliomas to the combination therapy resulted in a markedly increased survival benefit when compared to those exposed to either treatment alone [100]. Tumors of these mice had the highest level of cytotoxic T-cells and the lowest extent of regulatory T-cell infiltration among all groups.

Local RT and systemic PD-L1 blockade augment T-cell responses not only in the primary tumor but also at distant sites [67]. This refers to the abscopal effect that was first described in the 1950s by Mole et al. [101]. It is defined as the regression or disappearance of lesions outside of the irradiated field. This phenomenon has rarely been observed in routine clinical practice following the administration of RT alone [102]. With the advent of immune modifiers, however, the abscopal effect has been increasingly reported in preclinical models since radiation-induced systemic abscopal responses can be facilitated with additional immune manipulation [67, 99, 103–110].

Combining RT with anti-PD-1 antibody treatment has consistently produced abscopal effects on secondary tumors that are distant from the irradiated primary site in mouse models of melanoma, colon cancer, RCC, and breast cancer [99, 107]. Interestingly, a study done by Demaria et al. suggested that adding CTLA-4 blockade to RT (12Gy) in the treatment of 4T1 mice with primary mammary carcinomas inhibited the formation of lung metastasis [111]. A subsequent study from the same group combined a hypofractionated regimen (3 × 8Gy) with the anti-CTLA-4 therapy, and immune infiltrates and abscopal effects were found to be more pronounced than when either modality was used alone [108].

The timing of RT relative to immunotherapy may be another important consideration when combining RT with immunotherapy [95]. This question has not been addressed thoroughly in the preclinical models. In a study on a mouse model of breast cancer in which a combination of CTLA-4 blockade and RT was used, the antibody was administered at different time points with the best abscopal response being seen when the first dose of antibody was given during RT [108].

### 5.2. Mechanisms That Improve Antitumor Immune Responses and Abscopal Effects

*(1) Amelioration of Cancer-Cell Type I Interferon.* It is now established that the cyclic GMP-AMP Synthase (cGAS) - Stimulator of Interferon Genes (STING) pathway plays an important role in improving the antitumor immune response triggered by RT and immunotherapy. It induces IFN which is required to achieve optimal DC recruitment and cross-priming of effector T-cells [112–114]. Crosspresentation corresponds to the mechanism used by DCs to process and present tumor antigens to CD8+ T lymphocytes [115]. This is mainly mediated by a specialized subset of DCs that is dependent on the basic leucine zipper ATF-like transcription factor-3 (BATF-3) transcription factor and sp2/soluble FLT-3 ligand (sFLT-3L) growth factor for development [116]. Data suggests that this BATF-3 DC subset is essential for the therapeutic effects of anti-PD1 and anti-CD137 monoclonal antibodies by means of crosspresentation of tumor antigens [117].

Deng et al. demonstrated that these DCs activate the cGAS-STING signaling axis following the exposure of tumor cells to RT which results in the accumulation of irradiated tumor cell derived DNA. cGAS senses these RT-generated double-stranded DNA fragments and catalyzes the reaction between GTP and ATP responsible for the formation of the second messenger, cyclic GMP-AMP (cGAMP). The latter binds to the STING adaptor protein, triggering the phosphorylation of interferon regulatory factor 3 (IRF-3) by TANK-binding kinase 1 (TBK-1). IRF-3 then transports to the nucleus where transcription of inflammatory genes is triggered and an increase in type I IFN and other immune modulatory molecules results. Type I IFNs induced by RT have been shown to mediate the antitumor immune response and increase the frequency of CD8+ T-cells in tumor-draining lymph nodes [118].

In a preclinical study on mice bearing a B16 melanoma, those that were cGAS-deficient had a lower response to anti-PD-L1 treatment than wild-type controls [119]. cGAS knockout mice had lower numbers of tumor-specific CD4+ and CD8+ T-cells following immunotherapy when compared to wild-type ones. Interestingly, intramuscular injections of cGAMP in cGAS-deficient mice enhanced the effect of anti-PD-L1 treatment. 

*(2) Reduction of Tumor Microenvironment Immunosuppression.* Many studies show that the reduction in MDSC levels observed with the combination therapy might be critical in achieving an abscopal effect. In an experiment performed by Deng et al. on a mouse flank tumor model, substantial tumor regression was noted in the combination treatment group and was thought to be due to the concomitant increase in cytotoxic T-cell infiltration and the dramatic reduction in MDSCs [67]. In a patient receiving both, anti-CTLA-4 agents and palliative RT, an abscopal regression of a distant unirradiated tumor was immediately preceded by a sharp reduction in the proportion of MDSCs in the peripheral blood mononuclear cell population [109]. Demaria et al. suggested that the abscopal effect is an immune-driven phenomenon caused by T-cells within the irradiated tumor microenvironment [103]. RT primes antitumor cytotoxic T-cells that are usually unable to overcome suppressive effects of the tumor microenvironment without the help of immune modulators. Anti-CTLA-4 and anti-PD-L1 work through separate mechanisms to liberate T-cells from immunosuppression and drive the immune response [33, 120]. Kaminski et al. postulated that cytokines released by these activated T-cells have a major role in generating an abscopal effect [121]. For instance, tumor necrosis factor (TNF) is responsible for the direct elimination of MDSCs both, locally and systemically. In vitro cytotoxicity assays correlate TNF release by activated cytotoxic T-cells with apoptosis of MDSCs [67]. In addition, immunofluorescence staining studies performed on tumors treated with a combination of RT and anti-PDL1 show that MDSCs stained positive for cleaved caspase 3, an apoptosis marker [67]. This interaction between cytotoxic T-cells, TNF, and MDSCs has been verified in in vivo studies in which the expression of exogenous TNF was abrogated in mouse tumor models using an adenoviral vector [122, 123].

### 5.3. Dose Dependence of the Abscopal Effect

In an attempt to elicit greater antigen release and further improve the efficacy of immunotherapeutic agents, many preclinical studies tested the effect of combining them with hypofractionated regimens, particularly SBRT. The trials to date have used a variety of different doses and fractionations ranging from 15 to 75Gy in 1 to 15 fractions. The choice of optimal radiation dose and fractionation schema is related to the resulting abscopal effects.

A number of studies have demonstrated dose dependence of abscopal effect. Inferior abscopal effects have been noted with a single 20Gy dose of radiation as compared with regimens of 8Gy in three or 6Gy in five fractions [108, 124]. In a recent report in Nature Communications by Vanpouille-Box et al., mice with bilateral TSA tumors were exposed to RT to one tumor and were followed for responses in both irradiated and nonirradiated tumors [112]. In the absence of anti-CTLA-4, a single dose of 20 or 30Gy achieved comparable infield control to that of a regimen of 8Gy delivered in 3 fractions; however, only mice treated with 8Gy in 3 fractions were found to achieve abscopal responses and complete durable regression of their irradiated tumors upon the addition of anti-CTLA4. Of note, such responses were abrogated upon the depletion of CD8+ T-cells.

Gene expression analysis of cells from the irradiated tumors revealed an IFN type I gene signature following exposure to 8Gy in 3 fractions but not following a single dose of 20 or 30Gy [108]. This work explains the dependence of the abscopal effect on dose size and fractionation. Interestingly, several studies suggest a link between the cGAS-STING axis, RT dose per fraction, and RT's synergy with immunotherapy. In multiple murine and human carcinoma cells tested, cytosolic double-stranded DNA accumulated with increasing dose size per fraction up to a critical threshold of 10 to 12Gy. After this cutoff value, the abscopal effect rapidly decreased. Single doses in excess of 10 to 12Gy were found to induce Trex1, the exonuclease that degrades cytoplasmic DNA, thus precluding the activation of the cGAS/STING pathway [112]. In this way, IFN induction does not occur resulting in the absence of RT-induced abscopal effects with doses beyond 10 to 12Gy per fraction [125]. This leads to a decreased synergy between RT and immunotherapy [112, 126]. The work of Vanpouille-Box et al. proposed the challenge of delivering sufficient dose per fraction to generate enough dsDNA to trigger the cGAS/STING pathway, while at the same time preventing Trex1 induction [127]. This opens a new chapter in the debate of the choice of optimal dose and fractionation [128].

### 5.4. Safety and Efficacy of the Combination Therapy

The promising preclinical data on the combination therapy in mouse tumor models have resulted in a number of analyses reporting the safety and efficacy of this strategy in humans. In a retrospective study by Hubbeling et al., no significant difference was reported between RT-related adverse events observed in metastatic NSCLC patients who received prophylactic cranial RT combined with PD-1/PD-L1 inhibitors as compared to events observed in patients who received only cranial RT [129]. Park et al. demonstrated that in the clinical setting, RT and anti-PD-1 treatment resulted in a near complete regression of the primary tumors and a 66% reduction in distant tumors via abscopal responses [107]. Similarly, Postow et al. reported that palliative RT (28.5Gy in three fractions delivered over 7 days) given concurrently with maintenance Ipilimumab treatment in a patient with melanoma caused regression of the targeted lesion as well as marked abscopal effects [109].

To examine the feasibility and efficacy of RT combined with immune checkpoint blockade, several studies have been conducted as summarized in Table 1. In a phase I clinical trial of 22 patients with multiple melanoma metastases [33], a single lesion was irradiated with 6 to 8Gy delivered over two or three fractions, followed 3-5 days later by four cycles of Ipilimumab. Evaluation of the nonirradiated lesions by CT imaging using Response Evaluation Criteria in Solid Tumors (RECIST) demonstrated that 18% of patients had a PR as best response, 18% had SD, and 64% had PD. The median PFS and OS at median follow-up of 18.4 and 21.3 months were 3.8 and 10.7 months, respectively.

In another prospective clinical trial, 22 patients with stage IV melanoma were exposed to palliative RT (8Gy in 3 fractions or 4Gy in 10 fractions) five days following treatment with 4 cycles of Ipilimumab [34]. RT to 1-2 disease sites were initiated within 5 days after starting Ipilimumab. Patients had ≥1 nonirradiated metastasis measuring ≥1.5 cm for response assessment. Combination therapy was well tolerated without unexpected toxicities. Eleven patients (50.0%) had clinical benefit from therapy at median follow-up of 55 weeks, with 14% of them having CR, 14% having PR, and 23% having SD.

In another clinical trial by Tang et al., 35 patients were treated with SBRT (12Gy in 4 fractions or 6Gy in 10 fractions) either concurrently (1 day after the first dose of Ipilimumab) or sequentially (1 week after the second dose) [35]. Among 35 patients who initiated Ipilimumab, response outside the radiation field was assessable in 31 patients. Three patients (10%) exhibited PR, seven patients (23%) had SD lasting ≥6 months, and none had CR. Of note, clinical benefit was associated with increases in CD8+/CD4+ T-cell ratio, peripheral CD8+ T-cells, and proportion of CD8+ T-cells expressing PD1.

One of the largest prospective phase I studies (Abstract 20) to determine the safety and efficacy of SBRT in combination with Pembrolizumab in patients with metastatic solid tumors who progressed on standard treatment included doses ranging from 30Gy in 3 fractions to 50Gy in 5 fractions. Pembrolizumab therapy was initiated 7 days after the final SBRT treatment. According to data presented at the 2018 ASCO-SITC Clinical Immune-Oncology Symposium, SBRT prior to Pembrolizumab treatment was well tolerated, and the OR rate was 13.5% in the 68 patients who had imaging follow-up. Some abscopal responses were seen, whereby 26.9% of patients had a reduction of at least 30.0% in any single nonirradiated lesion and 13.5% of patients had a reduction of at least 30.0% in the aggregate sum of nonirradiated lesions [36]. A phase III trial (CA184-043) evaluated the use of RT followed by Ipilimumab or placebo in metastatic castration-resistant prostate cancer cases who progressed on Docetaxel chemotherapy. Post hoc analyses of subgroups revealed a trend toward improved OS in the Ipilimumab study arm with a p value of 0.053 [130].

Currently, at least 12 ongoing prospective clinical trials are evaluating the safety and efficacy of the combination of RT and immunotherapy in metastatic NSCLC [131]. Similarly, there are at least 21 clinical trials investigating the combination of RT with other immune-stimulating agents in pancreatic adenocarcinoma [132]. The results of these clinical trials, expected in the next few years, will greatly enhance our understanding of the potential for SBRT to synergize with ICIs to provide clinically meaningful improvements in patient outcomes [131].

As such, current data related to combination therapy in primary liver tumors are based on results from either preclinical animal models, which are inherently limited in their applicability to the clinical setting, or preliminary results from ongoing trials. Although results seem promising, implementation in clinical practice would be premature, as robust hypothesis-testing clinical trials are required to determine appropriate approaches of integrating these modalities.

## 6. Conclusion

While SBRT alone and immunotherapy alone have shown promise as effective therapies in patients with primary liver tumors, the combination of SBRT and PD-L1, PD-1, or CTLA-4 blockade has not been tested in these tumors [133]. It is expected that such an approach would result in improved therapeutic outcomes similar to those obtained in metastatic solid tumors, including melanomas and NSCLC. Many questions remain with regard to the optimal way to harness ionizing radiation in combination with immunotherapy, and how to best select patients for this approach [134]. We look forward to the results of the clinical trials presented in this review in hopes that outcomes can be improved for primary liver tumors.

## Figures and Tables

**Table 1:** List of studies on the combination of SBRT and immunotherapy in many cancers.

Author	Disease	N	RT	ICI	Schedule	Abscopal Effects
Twyman Nature [33]	Melanoma	22	6Gy x 2-3 8Gy x 2-3 (One Site)	Ipilimumab 3mg/kg/3w x4	Ipilimumab 3-5 days after RT	PD: 64% SD: 18% PR: 18% CR: None

Hiniker IJROBP 2016 [34]	Melanoma	22	8Gy x 3 4Gy x 10 (1-2 Sites)	Ipilimumab 3mg/kg/3w x4	RT within 5 days of Ipilimumab	SD: 23% PR: 14% CR: 14%

Tang CI. Can Res 2017 [35]	NSCLC, colorectal cancer (CRC), RCC, Others	35	12Gy x 4 6Gy x 10 (1 Site)	Ipilimumab 3mg/kg/3w x4	RT 1 day after Ipilimumab or 1 week after 2^nd^ Ipilimumab	PR: 10% SD: 23% CR: None

Luke JCO 2018 [36]	Ovarian, Endometrial, CRC, Others	73	30-50Gy (3-5, 2-4 Sites)	Pembrolizumab 200mg/3 weeks until progression, death, or toxicity	Pembrolizumab 7 days after SBRT	PD: 38 SD: 21 PR: 8 CR: 1

## List of Abbreviations

APC: Antigen Presenting Cells
BATF-3: Basic Leucine Zipper ATF-like Transcription Factor-3
CRT: Calreticulin
CTP: Child-Turcotte-Pugh
CCA: Cholangiocarcinoma
CR: Complete Response
cGAMP: Cyclic GMP-AMP
cGAS: Cyclic GMP-AMP Synthase
CTLA-4: Cytotoxic T-lymphocyte Associated Antigen-4
DC: Dendritic Cell
EBRT: External-Beam Radiation Therapy
EHC: Extrahepatic Cholangiocarcinoma
FFLP: Freedom from Local Progression
HCC: Hepatocellular Carcinoma
ICI: Immune Checkpoint Inhibitor
IFN: Interferon
IRF-3: Interferon Regulatory Factor 3
IHC: Intrahepatic Cholangiocarcinoma
LC: Local Control
MHC: Major Histocompatibility Complex
MWA: Microwave Ablation
MMR: Mismatch-Repair
MDSC: Myeloid Derived Suppressor Cell
NSCLC: Nonsmall-Cell Lung Cancer
OR: Objective Response
OS: Overall Survival
PR: Partial Response
PEI: Percutaneous Ethanol Injection
PD-1: Programmed-Death-1
PD-L1: Programmed-Death Ligand-1
RILD: Radiation-Induced Liver Disease
RFA: Radiofrequency Ablation
RT: Radiotherapy
RTPCR: Real-Time Polymerase Chain Reaction
RCC: Renal Cell Carcinoma
RECIST: Response Evaluation Criteria in Solid Tumors
STAT-3: Signal Transducer and Activator of Transcription-3
sFLT-3L: Sp2/Soluble FLT-3 Ligand
SD: Stable Disease
SBRT: Stereotactic Body Radiation Therapy
SRS: Stereotactic Radiation Surgery
STING: Stimulator of Interferon Genes
SEER: Surveillance, Epidemiology, and End Results
TBK-1: TANK-Binding Kinase 1
TACE: Transarterial Chemoembolization
TAA: Tumor-Associated Antigens
TIL: Tumor-Infiltrating Lymphocyte
TNF: Tumor Necrosis Factor
TKI: Tyrosine Kinase Inhibitor.
